# Temporal Effects of Bariatric Surgery on Adipokines, Inflammation and Oxidative Stress in Subjects with Impaired Glucose Homeostasis at 4 Years of Follow-up

**DOI:** 10.1007/s11695-019-04377-3

**Published:** 2020-01-03

**Authors:** Thinzar Min, Sarah L Prior, Gareth Dunseath, Rachel Churm, Jonathan D Barry, Jeffrey W Stephens

**Affiliations:** 1 Diabetes Research Group, Grove Building, Swansea University Medical School, Singleton Campus, Swansea University, Swansea, SA2 8PP UK; 2 Department of Diabetes and Endocrinology, Neath Port Talbot Hospital, Swansea Bay UHB, Swansea, SA12 7BX UK; 3 Applied Sports Technology, Exercise and Medicine (A-STEM) Research Centre, Swansea University, Swansea, SA1 8EN UK; 4 Welsh Institute of Metabolic & Obesity Surgery, Morriston Hospital, Swansea Bay UHB, Swansea, SA6 6NL UK; 5 Department of Diabetes and Endocrinology, Morriston Hospital, Swansea Bay UHB, Swansea, SA6 6NL UK

**Keywords:** Adipokines, Cytokines, Inflammation, Oxidative stress, Bariatric surgery, Diabetes

## Abstract

**Background:**

Previous studies have examined changes in plasma markers of inflammation and oxidative stress up to 24 months following bariatric surgery, but there is limited evidence on the long-term effects of bariatric surgery.

**Objectives:**

To examine the effects of bariatric surgery on adipokines (adiponectin, leptin), inflammatory cytokines [C-reactive protein (CRP), interleukin-6 (IL-6), interleukin-10(IL-10)] and global plasma measures of oxidative stress [thiobarbituric acid reactive substances (TBARS) and total antioxidant status (TAOS) 1 and 6 months, and 4 years post-surgery in subjects with obesity and impaired glucose regulation.

**Methods:**

A prospective study comprising of 19 participants (13 females, mean age 50.4 ± 6.2 years, mean body mass index (BMI) 54 ± 14 kg/m^2^, 17 type 2 diabetes) undergoing bariatric surgery (10 sleeve gastrectomy, 6 biliopancreatic diversion, 2 Roux-en-Y gastric bypass and 1 laparoscopic adjustable gastric banding). Serial measurements of the above markers were made pre-operatively, 1 and 6 months and 4 years post-operatively.

**Results:**

Compared to pre-operative levels, significant decreases were seen 4 years post-operatively in CRP (11.4 vs 2.8 ng/mL, *p* < 0.001), IL-6 (8.0 vs 2.1 pg/mL, *p* < 0.001) and leptin (60.7 vs 32.1 pg/mL, *p* = 0.001). At 4 years, both fasting and 120 min TAOS significantly increased by 35% and 19% respectively. However, fasting and 120 min TBARS did not show any significant changes.

**Conclusion:**

To our knowledge, no other studies have described changes in inflammation and oxidative stress at 4 years following bariatric surgery. This study contributes to the current literature supporting the longer-term beneficial effect of bariatric surgery on chronic inflammation and oxidative stress.

## Introduction

Chronic systemic inflammation and increased oxidative stress play a role in the pathogenesis of obesity, type 2 diabetes (T2DM) and cardiovascular disease [1]. Plasma levels of adipokines such as adiponectin and leptin and inflammatory cytokines such as C-reactive protein (CRP), interleukin-6 (IL-6), interleukin-10 (IL-10) and tumour necrosis factor alpha (TNF-α) are influenced by adipose tissue mass [2, 3]. Bariatric surgery leads to a significant reduction in adipose tissue mass and subsequent improvement in systemic inflammation [4].

With respect to oxidative stress, there are conflicting reports in the available literature. Catoi et al. observed no change in global measures of oxidative stress; nitrite and nitrate (NOx), total oxidant status (TOS), total antioxidant response (TAR), and oxidative stress index (OSI), 6 months after sleeve gastrectomy (SG) [5]. However, Cabrera et al. demonstrated that plasma level of glutathione (GSH) and total radical antioxidant parameter (TRAP) was increased and plasma levels of superoxide dismutase (SOD) and malondialdehyde (MDA) were decreased in 20 patients with obesity 12 months after Roux-en-Y Gastric Bypass (RYGB) [6]. It is important to note that in most of the published studies examining inflammation or oxidative stress, the follow-up period was up to 2 years. There is limited literature available on the long-term effect of bariatric surgery on inflammation and no literature available on long-term effect of bariatric surgery on oxidative stress.

Our previous work demonstrated that SG was associated with improvement in inflammation and oxidative stress in 55 participants with impaired glucose regulation [7]. The primary aim of the current study was to examine the long-term (4 years) effects of bariatric surgery on adipokines (adiponectin, leptin), inflammatory cytokines (CRP, IL-6, IL-10) and global plasma measures of oxidative stress (thiobarbituric acid reactive substances [TBARS] and total antioxidant status [TAOS]) regardless of operation types. The secondary aim was to investigate changes in adipokines (adiponectin, leptin), inflammatory cytokines (CRP, IL-6, IL-10) and global plasma measures of oxidative stress at 4 years in the SG group.

## Methods

### Study Participants

Approval for the study was obtained from the Local Research Ethics Committee (LREC reference 06/WMW02/7). Participants (*n* = 49) who took part in a previously published initial study (pre-operatively and followed 1- and 6-month post-operatively) were invited (by post, email or telephone call) to return at a median follow-up of 4 years (range 2–7 years). Of these, 19 agreed to participate. Twenty-four potential participants had relocated, declined or were not contactable, and 6 other potential participants were deceased. The study sample has been previously described [8]. In brief, the inclusion criteria at the outset of the previous study included males and females aged between 20 and 60 years with a body mass index (BMI) ≥ 40 kg/m^2^. All participants had previously diagnosed T2DM or diagnosed during an oral glucose tolerance test (OGTT) at the start of study, or impaired glucose regulation according to the American Diabetes Association (ADA) criteria [9]. Buse’s consensus (2009) criteria were used to define diabetes remission [10].

### Study Design

All participants underwent a standardised 75 g OGTT (122 mls of Polycal 61.9 g/100 ml of glucose, Nutricia Clinical Care, Trowbridge, UK) pre-operatively and at 1 month, 6 months and 4 years post-operatively. All participants were asked to fast from midnight before the test and all diabetes related medications were omitted for 24 h before the OGTT. Plasma samples for adiponectin, leptin, CRP, IL-6 and IL-10 were collected in the fasted state prior to the OGTT and samples for the measurement of TBARS and TAOS were collected in the fasted state and at 120 min afterwards. All samples were collected on ice, centrifuged within 1 h of collection and stored at − 80 °C until analysis. At the time of each OGTT, clinical and biochemical information were also obtained. Clinical measurements included weight, height, BMI, waist circumference and blood pressure. Biochemical data such as HbA1c and lipid profile were analysed within the local hospital accredited laboratory. Insulin was measured using an Invitron Insulin ELISA kit and C-peptide with an Invitron C-peptide luminescent kit. Insulin resistance and sensitivity were measured with Homeostasis Model Assessment (HOMA-2 model) by using measurements of fasting glucose and C-peptide concentrations [11]. These were calculated by using the Oxford University online calculator (https://www.dtu.ox.ac.uk/homacalculator/).

### Measurement of Inflammatory Cytokines

Plasma levels of adiponectin, leptin, CRP, IL-6 and IL-10 were measured with R&D Systems Quantikine ELISA kits. The intra- and inter-assay coefficients of variation of these immunoassays were as follows: adiponectin 4.7% and 6.9%, leptin 3.3% and 5.4%, CRP 8.3% and 7.0%, IL-6 4.2% and 6.4% and IL-10 5.0% and 7.5%.

### Measurement of Plasma TBARS and TAOS

MDA concentration, as a product of lipid peroxidation, was measured using a commercially available TBARS Assay (Cayman Chemical, MI, USA). A higher concentration of MDA is indicative of higher levels of lipid peroxidation, and therefore, higher oxidative stress within the sample. Intra- and inter-assay variability coefficients were 5.2% and 16.2% respectively. Plasma TAOS, which is inversely related to oxidative stress, was measured by Sampson’s modification of Laight’s photometric microassay [12]. Previous literature showed that plasma TAOS has a good correlation with plasma F2-isoprostanes [13]. The TAOS of plasma was determined by its capacity to inhibit the peroxidase-mediated formation of the 2,2-azino-bis-3-ethylbensthiazoline-6-sulfonic acid (ABTS+) radical. The difference in absorbance (control [saline] minus test [plasma]) divided by the control absorbance (expressed as a percentage) was used to represent the percentage inhibition of the reaction. Intra- and inter-assay variability coefficients were 4.3% and 10.1% respectively. Since ROS are highly reactive, they are difficult to measure in any biological sample, especially in easily accessible specimens such as serum or plasma. There are controversial views on whether oxidative stress measurements should be performed in a static fasting sample or following a dynamic testing such as with a glucose load [14]. Measuring plasma levels in the presence of a pro-oxidant stimulus such as glucose may provide a better measure of the dynamic response within an ex-vivo sample [15].

### Statistical Analysis

Statistical analysis was performed using SPSS (Version 22, SPSS Inc., Chicago). The normality of data was assessed by the Shapiro-Wilk test. Continuous data with a normal distribution are presented as mean and standard deviation, and data that did not have a normal distribution are described with the median and interquartile range. Log transformation of data was done where appropriate. We used ANOVA with post hoc analyses for data with a normal distribution and Friedman test for data without a normal distribution. The Pearson’s (normal distribution) correlation and the Spearman’s (non-parametric) correlation were used. In all cases, *p* < 0.05 was considered to be statistically significant.

## Results

### Participants’ Characteristics

Nineteen participants (13 females) with a mean age of 50.4 ± 6.2 years were included in the analysis. Of the 19 participants, 17 (89.5%) had T2DM and two had impaired fasting glucose (IFG) pre-operatively. The mean duration of T2DM was 37.7 ± 35.1 months. Ten underwent SG, 6 biliopancreatic diversion (BPD), 2 RYGB and 1 laparoscopic adjustable gastric banding (LAGB). The baseline characteristics along with the changes in anthropometric and clinical measures are summarised in Table 1. A significant decrease in body weight and BMI were observed 6 months [(43.2 ± 19.0% excess weight loss (EWL)] post-operatively and this was maintained at 4 years (43.7 ± 24.5 %EWL). Along with this, a significant decrease in diastolic blood pressure and a significant increase in HDL-C were observed. HbA1c also showed a significant reduction of 12 mmol/mol (20.7%) from baseline and 3 out of 17 subjects (17.6%) had achieved complete diabetes remission at 4 years.

**Table 1:** Changes in weight, blood pressure, lipid profile and glycaemic control

	Baseline	1 month	6 months	4 years	*p*
Weight (kg)	150 ± 37	132 ± 32	117 ± 29	116 ± 27	< 0.001
BMI (kg/m^2^)	54 ± 14	48 ± 12	43 ± 11	43 ± 11	< 0.001
%EWL		23.6 ± 10.3	43.2 ± 19.0	43.7 ± 24.5	
SBP (mmHg)	137 ± 25	123 ± 15	131 ± 14	129 ± 20	0.021
DBP (mmHg)	81 ± 14	71 ± 10	76 ± 8	73 ± 13	0.001
TC (mmol/L)	4.3 ± 0.8	3.8 ± 1.1	4.2 ± 1.3	4.6 ± 1.3	0.178
LDL (mmol/L)	2.3 ± 0.6	2.1 ± 0.9	2.5 ± 1.2	2.4 ± 1.0	0.416
HDL (mmol/L)	1.2 ± 0.3	1.1 ± 0.3	1.2 ± 03	1.5 ± 0.6	0.002
TG (mmol/L)	1.7 ± 0.9	1.5 ± 0.5	1.4 ± 0.5	1.3 ± 0.5	0.151
FPG* (mmol/L)	7.3 (5.9–9.2)	5.7 (4.8–6.8)	5.4 (4.5–6.9)	6.4 (5.5–9.0)	0.378
2 h-PG (mmol/L)	13.1 ± 5.7	8.8 ± 3.8	9.2 ± 6.6	9.4 ± 5.5	0.238
HbA1c (mmol/mol)	58 ± 18	47 ± 12	45 ± 15	46 ± 15	0.049
Fasting insulin* (mU/L)	27.7 (19.6–38.6)	12.6 (7.6–25.6)	10.3 (6.0–20.7)	16.9 (6.0–34.4)	0.135
Fasting C-peptide* (pmol/ml)	1.4 (0.9–1.6)	1.2 (0.8–1.6)	0.8 (0.6–1.1)	0.5 (0.3–0.7)	0.018
HOMA-%S**	1.6 ± 0.3	1.7 ± 0.3	1.8 ± 0.5	1.8 ± 0.4	0.064
HOMA-IR**	0.4 ± 0.2	0.2 ± 0.3	0.1 ± 0.4	0.2 ± 0.4	0.098

Data are presented as mean ± SD unless otherwise stated.*Data are presented as median and interquartile range. **Log transformed data. *%EWL*, percent excess weight loss; *SBP*, systolic blood pressure; *DBP*, diastolic blood pressure; *TC*, total cholesterol; *LDL*, low density lipoprotein cholesterol; *HDL*, high density lipoprotein cholesterol; *TG*, triglyceride. *FPG*, fasting plasma glucose; *2 h-PG*, 2-h plasma glucose; *HbA1c*, haemoglobin A1c; *HOMA-%S*, homeostatic model assessment (insulin sensitivity); *HOMA-IR*, homeostatic model assessment (insulin resistance)

*p* value calculated from ANOVA or Friedman test

### Changes in Adipokines and Inflammatory Cytokines

Table 2 shows temporal changes in adipokines and inflammatory cytokines. At 4 years, adiponectin did not show any change compared to the pre-operative levels. Plasma leptin concentration showed a linear decrease from 60.7 pg/mL pre-operatively to 40.4 pg/mL at 1 month, 33.7 pg/mL at 6 months and 32.1 pg/mL at 4 years (*p* = 0.001). The median plasma CRP concentration decreased from 11.4 ng/mL pre-operative to 3.7 ng/mL at 1 month, 5.8 ng/mL at 6 months and 2.8 ng/mL at 4 years (*p* = 0.003). IL-6 was significantly decreased at 4 years compared to the pre-operative value (73.8% decrease, *p* = 0.009). However, IL-10 levels did not show any significant change compared to the pre-operative levels.

**Table 2:** Changes in adipokines, inflammatory cytokines and markers of oxidative stress

	Baseline	1 month	6 months	4 years	*p*
Adiponectin* (ng/mL)	8.3 (5.3–10.5)	7.7 (4.0–12.2)	8.2 (4.6–12.1)	6.3 (4.7–13.3)	0.937
Leptin* (pg/mL)	60.7 (32.3–70.3)	40.4 (23.1–62.8)	33.7 (15.6–51.7)	32.1 (15.4–45.7)	0.001
CRP* (ng/mL)	11.4 (3.5–33.0)	3.7 (1.9–19.3)	5.8 (1.9–32.1)	2.8 (1.5–5.7)	0.006
IL-6* (pg/mL)	8.0 (4.2–16.7)	5.6 (3.8–23.3)	11.7 (5.1–24.9)	2.1 (0.9–5.4)	0.005
IL-10* (pg/mL)	7.2 (2.5–9.3)	6.4 (4.9–9.4)	7.1 (5.2–9.4)	5.1 (3.9–8.4)	0.140
Fasting TBARS^1^ (ng/mL)	44 ± 29	24 ± 16	26 ± 8	63 ± 20	0.388
120-min TBARS^2^ (ng/mL)	74 ± 41	49 ± 24	43 ± 24	72 ± 24	0.157
Fasting TAOS (%)	38.3 ± 10.8	35.5 ± 9.1	36.3 ± 8.5	51.6 ± 17.2	0.042
120-min TAOS (%)	40.9 ± 7.6	39.4 ± 9.3	33.6 ± 10.8	48.5 ± 13.5	0.026

*Data are presented as median and interquartile range. Data are presented as mean and SD. ^1^Nine outliers excluded from analysis. ^2^Eight outliers excluded from the analysis. *p* value calculated from ANOVA or Friedman test

### Changes in Plasma Markers of Oxidative Stress

A non-significant increase in fasting TBARS, and no change in 120-min TBARS were noted at 4 years compared to the baseline pre-operative values (Table 2). A non-significant reduction in fasting and 120-min TBARS was observed at both 1 and 6 months. Compared to the pre-operative values, fasting TAOS increased by 35% (*p* = 0.011) and 120-min TAOS increased by 19% (*p* = 0.048) at 4 years after surgery (Table 2).

### Correlations Between Changes (Delta Δ) from Pre-operative to 4 Years Post-operative in Weight, Glucose, Insulin Resistance, Adipokines, Inflammatory Cytokines and Markers of Oxidative Stress

There were significant positive correlations between Δleptin and Δweight (*r* = 0.656, *p* = 0.002), and ΔBMI (*r* = 0.734, *p* = 0.001), suggesting a greater reduction in weight was associated with a greater reduction in leptin. Changes in 120-min TAOS had negative correlation with Δ fasting plasma glucose (FPG) (*r*_s_ = − 0.66, *p* = 0.004); and 120-min TBARS had positive correlation with HbA1c (*r* = 0.506, *p* = 0.045). No other correlations were observed between weight, glucose, insulin resistance, adiponectin and inflammatory cytokines.

### Sub-group Analysis: the SG Group

The SG group had a mean age of 52 ± 6 years and a mean BMI of 48.4 ± 7.2 kg/m^2^. Changes in clinical measures of obesity, glucose metabolism and incretin hormones in this cohort were recently published [16]. In brief, a significant reduction in weight, BMI and markers of glycaemia and a significant increase in total cholesterol, HDL cholesterol and LDL cholesterol were observed at 4 years. There was no significant change in adiponectin levels but a significant reduction in leptin by 54% at 4 years compared to baseline (Table 3). Similarly, CRP decreased by 64% and IL-6 decreased by 84%. With regard to markers of oxidative stress, a significant increase in fasting TBARS and fasting TAOS but no significant changes in 120-min TBARS and TAOS were observed (Table 3).

**Table 3:** Adipokines, inflammatory cytokines and markers of oxidative stress before and 4 years after SG

	Baseline	4 years	*p*
Adiponectin (ng/mL)	8.5 (5.8–12.7)	9.1 (5.1–13.3)	0.721
Leptin (pg/mL)	49.1 (30.8–63.9)	27.5 (13.0–44.1)	0.037
CRP (ng/mL)	7.2 (3.2–20.0)	2.6 (1.5–7.6)	0.007
IL-6 (pg/mL)	8.1 (4.9–38.8)	1.3 (0.4–4.6)	0.005
IL-10 (pg/mL)	8.4 (3.5–13.9)	4.9 (3.5–6.5)	0.093
Fasting TBARS (ng/mL)	54 (30–84)	125 (91–208)	0.021
120-min TBARS (ng/mL)	85 (44–132)	93 (80–160)	0.859
Fasting TAOS (%)	38 (47–52)	59 (43–63)	0.038
120-min TAOS (%)	44 (36–46)	50 (32–58)	0.374

Data are presented as median and interquartile range. *p* value calculated from Wilcoxon signed rank test

## Discussion

Bariatric surgery is associated with an improvement in metabolic outcomes and a reduction in cardiovascular risk factors. It is postulated that this is likely to be related to an improvement in the inflammatory environment as a result of rapid and substantial weight loss. In line with previously published studies, which had shorter follow-up duration (6 to 24 months) [4, 8, 17], we observed a significant reduction in pro-inflammatory biomarkers (leptin, CRP, IL-6) at 4 years. These improvements were accompanied by an improvement in body weight (44% EWL), an improvement in glycaemic control (65% achieving a target HbA1c ≤ 6.5%) and improvements in cardiovascular risk factors (diastolic blood pressure and HDL-C). However, we observed no significant change in plasma adiponectin level (but numerical decrease). One possible explanation for this unexpected finding of plasma adiponectin level could be type 2 error and the other explanation might be due to small sample size.

Of note, two previous studies have examined changes in cytokines at 3 years following bariatric surgery. One study examined CRP, adiponectin, leptin, visfatin, IL-6 and TNF-α in 10 subjects with normal glucose tolerance following BPD. The authors reported that after considerable weight loss (53% EWL), leptin, CRP and IL-6 decreased but adiponectin increased significantly. No significant changes were observed in TNF-α [18]. Another study comprising of 28 subjects with the metabolic syndrome, examined changes in adipocytokines before and 3, 6, 12 and 24 months after vertical banded gastrectomy. Of these, six subjects had 36- and 48-month follow-up. The authors reported a linear increase in levels of adiponectin and resistin along with a non-significant decrease in CRP at 36 and 48 months, and a significant decrease in leptin at 36 months compared to baseline [19].

In the current study, there was a numerical reduction (30%) in IL-10 at 4 years compared to baseline. Although IL-10 is generally regarded as an anti-inflammatory cytokine, some human experimental studies revealed pro-inflammatory property of IL-10 [20]. The published literature contains conflicting reports relating to the effect of bariatric surgery on IL-10. Netto et al. demonstrated that IL-10 increased by 123 ± 55% (*p* = 0.02) in 41 subjects with obesity without T2DM, 6 months after RYGB [21]. Mallipedhi et al. showed no significant changes in IL-10 concentration in 22 patients with T2DM and impaired glucose regulation 6 months after SG [8].

With respect to oxidative stress, fasting and 120-min TAOS significantly increased at 4 years compared to the pre-operative levels. TAOS, a global measure of antioxidant status, is inversely associated with oxidative stress and obesity [22]. Previous work by Prior et al. observed no change in TAOS in 22 participants with morbid obesity and impaired glucose regulation 6 months following SG [14]. Two other studies also reported no changes in plasma TAOS following surgical induced weight loss. Catoi et al., examined TAOS and total antioxidant response (TAR) in patients with morbid obesity in comparison with a normal weight control group (*n* = 23 each group). No significant changes were observed in TAOS 12 months after silastic ring vertical gastroplasty [23]. Melissa et al. described no change in TAOS in 16 subjects with obesity, 6 months following intragastric balloon, despite significant reduction in body weight [22].

It is important to note that TAOS can be influenced by many factors such as age [24], diet [25], vitamin supplementation [26] and physical activity [27]. Possible explanations for an increase in TAOS within the current study include changes in dietary habit following bariatric surgery; vitamin supplementations following bariatric surgery; possible changes in physical activity due to weight loss; a decrease in pro-inflammatory cytokines; a better glycaemic control or an improvement in cardiovascular risk factors.

Within the current study, no significant change in TBARS was observed at 4 years, compared to the pre-operative values. However, we observed an initial decrease (but not statistically significant) in TBARS at 1 month and 6 months. There are inconsistent observations on the effect of bariatric surgery on TBARS. Uzun et al. showed a significant decrease in MDA and oxidized LDL (ox-LDL) and a strong positive relationship between MDA and BMI (*r* = 0.79, *p* < 0.001) in 20 subjects with obesity, 6 months after LAGB [28]. Boesing et al. observed that RYGB was associated with an increase in TBARS, despite a significant weight loss in 20 subjects at 6 months [29]. On the other hand, Dadalt et al. (*n* = 35) described an initial decrease in TBARS at 12 months after RYGB but this was not preserved at 24 months, when 25.7% of the participants regained weight [30].

We observed a positive correlation between Δleptin with both Δweight and ΔBMI, suggesting a change in weight was associated with a change in leptin. Leptin is an orexigenic hormone and high serum leptin concentrations are associated with high BMI and body fat mass [31]. We also observed a negative correlation between Δ120-minute TAOS with ΔFPG and a positive correlation between Δ120 minutes TBARS with ΔHbA1c. These findings were in agreement with previous studies describing an association between oxidative stress and markers of glycaemia [32].

In the exploratory sub-group analysis, we demonstrated that SG was associated with a significant reduction in pro-inflammatory biomarkers (leptin, CRP, IL-6) along with an improvement in body weight and glycaemic control at 4 years. Our findings were in agreement with previous studies. Zhu et al. demonstrated a reduction in leptin, CRP and IL-6 in patients with morbid obesity and subclinical hypothyroidism 12 months following SG [33]. Similarly, Salman et al. demonstrated that the levels of serum adiponectin significantly increased, while the levels of serum leptin, resistin, CRP, plasminogen activator inhibitor-1 and serum amyloid-A significantly decreased at 6 months after SG [34]. In contrast to previous studies [18, 19, 34], we observed no statistically significant change in serum adiponectin levels. This might be explained by a small sample size. In our recent published study comprising 55 participants with impaired glucose regulation, we observed a significant increase in serum adiponectin level at 6 months following SG [7]. Within the SG group, there was a significant increase in fasting TBARS as well as fasting TAOS. Since TBARS is a measure of lipid peroxidation, an increase in total cholesterol and LDL cholesterol following SG might explain an increase in fasting TBARS. Previous studies have shown that SG has no significant impact on lowering total cholesterol and LDL-C but is associated with an increase in HDL-C [35–37]. An increase in TAOS reflects a reduction in global oxidative stress and an improvement in metabolic profile. Of note, plasma TAOS is correlated positively with HDL-C and negatively with glucose and HBA1c [13].

There are limitations to the current study. The first limitation was a small sample size and a considerable drop-out at 4 years. In addition, a considerable number (*n* = 8) of participants were excluded for analysis of TBARS due to haemolysis of blood sample. The second limitation was that the sample comprised of three participants who had impaired glucose tolerance, since the level of chronic inflammation and oxidative stress might differ between those with established T2DM. The third limitation was the accuracy and variability of duration of diabetes within the group (mean duration 37 ± 35 months) and this might influence the level of inflammatory makers and oxidative stress pre-operatively. The fourth limitation was that factors influencing antioxidant status such as diet history, physical activity and uric acid concentrations were also not recorded. The fifth limitation was the heterogeneity of bariatric procedure. However, previous studies demonstrated that weight lost is not dissimilar amongst SG, RYGB and BPD [38]. Our aim was to examine long-term (4 years) changes in inflammatory biomarkers and oxidative stress following bariatric surgery irrespective of surgical technique. Since the SG was the predominate procedure in this cohort, we also reported sub-group exploratory analysis for the SG group. To our knowledge, no other studies have described changes in inflammation and oxidative stress at 4 years following bariatric surgery. The current study contributes to the current literature supporting the beneficial effect of bariatric surgery on chronic inflammation and oxidative stress at 4 years. To translate our findings into clinical setting, larger studies with adequate power and long-term follow-up are warranted.
